# Use of antipsychotics in patients with epilepsy: a case report

**DOI:** 10.1192/j.eurpsy.2025.2246

**Published:** 2025-08-26

**Authors:** P. Perez- Melendez Perez, H. Vizcaino Herrezuelo, C. Delgado Marmisa

**Affiliations:** 1 Psychiatry Department, Hospital Universitario Puerta de Hierro, Madrid, Spain

## Abstract

**Introduction:**

The use of antipsychotic medications in individuals with epilepsy has been studied extensively. Starting neuroleptic therapy in patients with epilepsy is complicated due to the potential for these drugs to lower the seizure threshold. Consequently, both psychiatrists and neurologists must collaborate to develop personalized treatment plans for these patients.

**Objectives:**

To evaluate various therapeutic options for patients experiencing both psychotic symptoms and seizures, aiming to select the most appropriate treatment for each individual.

**Methods:**

This case report describes a 47-year-old male patient who is presented with a diagnosis of symptomatic focal epilepsy in the left temporoparietal malacic area post traumatic brain injury. Following a traffic accident at the age of 14, after which he suffered a temporoparietal hematoma, the patient has presented numerous epileptic seizures. Initially, he abandoned treatment and follow-up with neurology, which he resumed in 2021. Additionally, the patient was referred to psychiatry after verbalizing delusional ideation of persecution with affective repercussions (tendency towards irritability) and behavioral repercussions (tendency towards social isolation and difficulties in the work environment) as well as auditory hallucinations with derogatory content. Neurology initiated treatment with eslicarbazepine 800mg, with cessation of epileptic seizures. After considering different treatment options and taking into account interactions with antiepileptic treatment, risperidone 1mg was initiated.

**Results:**

Following the initiation of risperidone, the patient experienced a reduction in irritability and has not presented further epileptic seizures. Due to potential drug interactions, the risperidone dose was gradually titrated upwards, resulting in a decrease in delusional ideation and improved overall functioning.

**Conclusions:**

Patients with epilepsy and comorbid psychotic symptoms require a multidisciplinary approach, including individualized treatment. Neuroleptic medications can significantly improve quality of life in these patients. Therefore, it is essential to carefully select the appropriate antipsychotic, starting at low doses and gradually titrating upwards, with close monitoring to ensure patient safety and drug efficacy.

**Disclosure of Interest:**

None Declared

